# Immobilization, Lymphedema, and Obesity are Predictive Factors in the Development of Adhesive Capsulitis in Breast Cancer Patients

**DOI:** 10.1055/s-0043-1772479

**Published:** 2023-11-09

**Authors:** Marcos Rassi Fernandes, Flaviane Marques de Assis, Joana Ermida Spagnol, Vinícius Barros Chaves

**Affiliations:** 1 Universidade Federal de Goiás, Goiânia, GO, Brazil

**Keywords:** breast cancer, shoulder, adhesive capsulitis, obesity, lymphedema, câncer de mama, ombro, capsulite adesiva, obesidade, linfedema

## Abstract

**Objective** Adhesive capsulitis is a condition characterized by shoulder pain and stiffness. Breast cancer treatment has been linked to the development of this condition, but its mechanisms are still little known. This study's objective was to identify predictors factors associated with the development of adhesive capsulitis in breast cancer patients.

**Methods** A case control study was performed with women undergoing treatment for breast cancer in a single center. The sampling was nonprobabilistic and consecutive. Adhesive capsulitis was defined as constant pain associated with decreased active and passive shoulder movement in anterior elevation, external rotation at 0°/90° abduction, and internal rotation at 90° abduction. The study group consisted of patients with shoulder pain and range of motion limitations, while the control group consisted of women without any shoulder abnormalities. Sociodemographic and clinical variables were collected. A univariate logistic regression was used to assess the influence of variables on the studied outcome. For *p* < 0.20, a multivariate logistic regression was used. The probability of null hypothesis rejection was 5%.

**Results** A total of 145 women were assessed, with 39 (26.9%) on the study group and 106 (73.1%) on the control group. The majority was under 60 years old. In the multivariate analysis, variables correlated to the outcome under study were shoulder immobilization (OR = 3.09; 95% CI: 1.33–7.18; *p* = 0.009), lymphedema (OR = 5.09; 95% CI: 1.81–14.35; *p* = 0.002), and obesity (OR = 3.91; 95% CI: 1.27–12.01; *p* = 0.017).

**Conclusion** Lymphedema, postsurgery immobilization, and obesity are predictive factors for the development of adhesive capsulitis in breast cancer patients.

## Resumo

**Objetivo** Capsulite adesiva é uma afecção caracterizada por dor e limitação dos movimentos do ombro. O tratamento do câncer de mama está relacionado ao desenvolvimento dessa doença por meio de mecanismos ainda pouco conhecidos. O objetivo do estudo foi identificar os fatores associados ao desenvolvimento de capsulite adesiva em pacientes com câncer de mama.

**Métodos** Um estudo caso-controle foi realizado com mulheres em tratamento para câncer de mama em um centro único. A amostra foi consecutiva e não-probabilística. A capsulite adesiva foi pré-definida como dor constante e diminuição da amplitude de movimentos em elevação anterior, rotação externa em 0°/90° abdução e rotação interna em 90° abdução. O grupo caso foi constituído por pacientes com dor e limitação de todos os movimentos do ombro, enquanto o controle por pacientes sem qualquer alteração nesta articulação. Variáveis sociodemográficas e clínicas foram coletadas. Foi realizada uma análise de regressão logística univariada para avaliar a influência das variáveis em relação ao desfecho estudado. Para valores de *p* < 0,20, realizou-se a análise de regressão logística multivariada. A probabilidade de se rejeitar a hipótese nula foi de 5%.

**Resultados** Foram avaliadas 145 mulheres, sendo 39 casos (26,9%) casos e 106 controles (73,1%). Na análise multivariada, as variáveis associadas ao desfecho estudado foram imobilização do ombro (OR = 3,09; 95% IC: 1,33–7,18; *p* = 0,009), linfedema (OR = 5,09; 95% IC: 1,81–14,35; *p* = 0,002) e obesidade (OR = 3,91; 95% IC: 1,27–12,01; *p* = 0,017).

**Conclusão** Linfedema, imobilização pós-cirúrgica e obesidade são fatores preditores associados ao desenvolvimento de capsulite adesiva em pacientes com câncer de mama.

## Introduction

Adhesive capsulitis (AC) is a condition that causes thickening of the glenohumeral joint capsule, along with constant pain and progressive loss of the shoulder's passive and active range of motion. 1 2 3 4 It is prevalent in approximately 2 to 5% of the overall population, especially women aged between 40 and 60 years old. 4 This condition may be classified as primary (idiopathic) or secondary to trauma, surgery, or other comorbidities, such as thyroid conditions, dyslipidemia, diabetes mellitus, Parkinson's, cardiopathies, and breast cancer (BC). 1 3 4 5

This type of cancer is highly prevalent worldwide, with 2.3 million new cases reported just in 2020. By 2040, BC is predicted to increase to over 3 million new cases and 1 million deaths every year. It is a heterogeneous condition, morphologically divided into in situ (cancerous cells limited to the basement membrane) and invasive (cancerous cells crossing the basement membrane) carcinoma. 6 There are several conditions that predispose to its development, such as older age, female sex, genetic mutations, previous history of ductal carcinoma in situ, high body mass index (BMI), nulliparity or first pregnancy after 30 years of age, early menarche, history of BC or ovarian cancer in the family, late menopause, exposure to previous thoracic radiation therapy, and slightly elevated combined hormone replacement therapy in postmenopause women. 7 8

Advancements in the diagnosis and treatment of BC have resulted in a higher survival rate at 5 years (89% in the USA), which has led to an increase in the number of shoulder morbidity cases. 9 10 11 One of such morbidities is AC, 5 9 which according to Yang et al. accounts for 10.3% of patients treated within a period of 13 to 18 months. 5

In this setting, despite BC having been established as risk factor for AC, predictive factors for this correlation have not been established. Thus, this study's objective is to identify factors associated with AC development in patients with BC. Our hypothesis is that more aggressive forms of cancer treatment, such as radiation therapy and mastectomy, in addition to immobilization of the shoulder, would favor the development of this condition.

## Methods

This is a case control study conducted in a single university hospital, specifically at the Advanced Breast Diagnostics Center (CORA), between September 2020 and March 2021. The study was approved by the Institutional Review Board on June 28, 2020, under the protocol number 4.119.154. All participants signed an informed consent form before data collection began.

About 160 women a month are seen at CORA for BC treatment and investigation. Yang et al. 5 reported AC prevalence in patients treated for BC at 10.3%. Considering the inclusion and exclusion criteria and assuming α error of 5% and β error of 20% (80% power), the calculated sample size was 145 subjects.

The sampling was nonprobabilistic and consecutive. We defined AC as constant pain associated with decreased active and passive shoulder movement in anterior elevation, external rotation at 0°/90° abduction, and internal rotation at 90° abduction. We did the diagnosis of AC in the BC ambulatory.

Patients over the age of 18 years old undergoing BC treatment and with history of it were included. Furthermore, patients with shoulder pain or limited range of motion before BC treatment, presenting with other BC predisposing conditions, locked dislocation of the shoulder, glenohumeral arthrosis, avascular necrosis of the humeral head, or previous history of trauma/surgery of the affected shoulder were excluded.

After excluding the aforementioned conditions, the case group consisted of BC patients presenting with AC at the time of enrollment, and the control group consisted of BC patients without AC.

Data were collected using a sociodemographic and clinical questionnaire. The questionnaire was applied by researchers individually in a private environment. Patients' charts were also revised to collect relevant data.

After the questionnaire, patients reporting at rest and in motion shoulder pain with the aid of the visual analogue scale for pain underwent physical examination to confirm the AC diagnosis. The diagnosis consisted of active and passive shoulder mobility assessment in dorsal decubitus and hip and knee flexion with a digital Kaptron goniometer model 360 (Kaptron, Porto Alegre, RS, Brazil).

A digital Toledo balance model 9091 (Toledo, São Bernardo do Campo, SP, Brazil), with a calibrated Filizola meter (São Paulo, SP, Brazil) was used to measure patients' weight and height for BMI calculation.

The outcome was AC diagnosis. Independent variables were collected with the applied questionnaire, which contained sociodemographic and clinical variables related to BC.

Sociodemographic variables were age (< or ≥ 60 years old); BMI (normal/overweight/obesity); race (white/nonwhite); marital status (married/divorced/widowed/single); monthly income (in minimum wages); level of education (illiterate/elementary school/middle school/highschool/higher education), and dominance (right or left-handed).

The relevant clinical data were: side (right/left/bilateral); biopsies (½ to 3/over 4); breastfeeding (yes/no); menopause (yes/no); previous hormone replacement therapy (yes/no); stage at time of diagnosis (early/advanced); chemotherapy (yes/no); radiation therapy (yes/no); endocrine therapy (yes/no); surgery (yes/no); and shoulder immobilization (yes/no). For patients undergoing mastectomy: type of mastectomy (quadrantectomy/mastectomy), lymph nodes dissection (no/sentinel/axillary emptying), and development of lymphedema after surgery (yes/no). For patients undergoing immobilization: time of immobilization (1–3 or ≥4 weeks).

Categorical variables were described with absolute (n) and relative frequency (%), and quantitative variables were described with average and standard deviation. Homogeneity among subjects in the study and control groups was analyzed with the Student *t* -test for continuous variables and the Pearson Chi-squared test for categorical variables. The univariate logistic regression analysis test was used to assess the influence of predictor variables for the development of AC, for variables with *p* < 0.20, a multivariate logistic regression analysis test was used (odds ratio, OR, and 95% confidence interval, CI). Excel 2007 (Microsoft Corp. Redmond, WA, USA) and the Statistical Package Social Sciences (SPSS, IBM Corp. Armonk, NY, USA), version 22.0 were used for tabulating data and statistical analysis, respectively.

## Results

A total of 175 patients with BC were recruited, but 30 were excluded due to presenting shoulder pain with a history of trauma or other joint conditions and without motion limitation. 145 patients remained, 39 of which were in the study group (26.9%) and 106 in the control one (73.1%). Please refer to Table 1 for BC patients' sociodemographic and clinical data.

**Table 1:** Breast cancer patients' sociodemographic and clinical data for case ( *n* = 39) and control ( *n* = 106) groups

Variables	Case n (%)	Control n (%)	Total n (%)	*p* *
Sociodemographic variables				
Age group				
< 60 years	33 (84.6)	72 (67.9)	105 (72.4)	0.059
≥ 60 years	6 (15.4)	34 (32.1)	40 (27.6)	
BMI				
Normal	6 (15.4)	40 (37.7)	46 (31.7)	0.018
Overweight	14 (35.9)	36 (34.0)	50 (34.5)	
Obesity	19 (48.7)	30 (28.3)	49 (33.8)	
Race				
White	16 (41.0)	36 (34.0)	52 (35.9)	0.432
Not white	23 (59.0)	70 (66.0)	93 (64.1)	
Marital status				
Married	18 (46.2)	48 (45.3)	66 (45.5)	0.971
Divorced	6 (15.4)	16 (15.1)	22 (15.2)	
Widow	4 (10.3)	14 (13.2)	18 (12.4)	
Single	11 (28.2)	28 (26.4)	39 (26.9)	
Monthly income				
None	2 (2.6)	10 (5.9)	12 (5.0)	0.259
Up to 1 MW	16 (42.1)	56 (54.9)	72 (51.4)	
2 or 3 MW	18 (47.4)	37 (36.3)	55 (39.3)	
≥ 4 MW	3 (7.9)	3 (2.9)	6 (4.3)	
Education				
Illiterate	1 (2.6)	5 (2.9)	6 (2.8)	0.234
Elementary school	5 (12.8)	22 (21.2)	27 (18.9)	
Middle school	6 (15.4)	16 (15.4)	22 (15.4)	
Highschool	26 (66.7)	51 (49.0)	77 (53.8)	
Higher	1 (2.6)	12 (11.5)	13 (9.1)	
Dominance				
Right-handed	36 (92.3)	101 (95.3)	137 (94.5)	0.487
Left-handed	3 (7.7)	5 (4.7)	8 (5.5)	
Clinical breast data				
Affected breast				
Right	18 (46.2)	52 (49.1)	70 (48.3)	0.618
Left	18 (46.2)	50 (47.2)	68 (46.9)	
Both	3 (7.7)	4 (3.8)	7 (4.8)	
Number of biopsies				
1	12 (30.8)	50 (47.2)	62 (42.8)	0.206
2 to 3	23(59.0)	47 (44.3)	70 (48.3)	
≥ 4	4 (10.3)	9 (8.5)	13 (9.0)	
Breastfeeding				
Yes	36 (92.3)	90 (84.9)	126 (86.9)	0.241
No	3 (7.7)	16 (15.1)	19 (13.1)	
Menopause				
Yes	30 (76.9)	82 (77.4)	112 (77.2)	0.956
No	9 (23.1)	24 (22.6)	33 (22.8)	
Chemotherapy				
Yes	33 (84.6)	89 (84.0)	122 (84.1)	0.924
No	6 (15.4)	17 (16.0)	23 (15.9)	
Radiotherapy				
Yes	35 (89.7)	71 (67.0)	106 (73.1)	0.006
No	4 (10.3)	35 (33.0)	39 (26.9)	
Endocrinotherapy				
Yes	25 (64.1)	65 (61.3)	90 (62.1)	0.760
No	14 (35.9)	41 (38.7)	55 (37.9)	
Previous hormone replacement therapy				
Yes	1 (2.6)	10 (9.4)	11 (7.6)	0.166
No	38 (97.4)	96 (90.6)	134 (92.4)	
Breast cancer stage				
Initial	23 (59.0)	60 (56.6)	83 (57.2)	0.798
Advanced	16 (41.0)	46 (43.4)	62 (42.8)	
Surgery				
Yes	38 (97.4)	80 (75.5)	118 (81.4)	0,003
No	1 (2.6)	26 (24.5)	27 (18.6)	
Type of surgery				
Quadrantectomy	18 (47.4)	39 (48.8)	57 (48.3)	0.567
Mastectomy	20 (52.6)	41 (51.3)	61 (51.7)	
Lymph node dissection				
No	5 (13.2)	15 (18.8)	20 (17.0)	0.537
Sentinel	9 (23.7)	23 (28.7)	32 (27.1)	
Axillary dissection	24 (63.2)	42 (52.5)	66 (55.9)	
Immobilization				
Yes	22 (56.4)	26 (24.5)	48 (33.1)	<0.001
No	17 (43.6)	80 (76.5)	97 (66.9)	
Immobilization time				
1 to 3 Weeks	8 (36.4)	11 (42.3)	19 (39.6)	0.675
≥ 4 Weeks	14 (63.6)	15 (57.7)	29 (60.4)	
Lymphedema				
Yes	13 (34.2)	8 (11.2)	21 (18.6)	0.001
No	25 (65.8)	72 (88.7)	97 (81.4)	

**Abbreviations:** BMI, body mass index; MW, minimum wage; n, absolute frequency; SD, standard deviation. **Notes:** * Pearson chi-square test for categorical variables.

For the case group, according to the visual analogic scale (VAS), the mean pain scores at rest and at motion were 4.21 and 7.38, respectively. The mean passive range of motion was 121.8° of anterior elevation, 53.4° and 53.5° of external rotation at 0°/90° of abduction, and 57.9° of internal rotation. Univariate analysis ( Table 2 ) showed the following predictor variables with *p* < 0.20: age group ≥60 years old (0.052); obesity ( *p* = 0.006); monthly income ≥4 minimum wages ( *p* = 0.153); previous hormonal replacement therapy ( *p* = 0.197); radiation therapy ( *p* = 0.010), surgery ( *p* = 0.015), immobilization ( *p* < 0.001), and lymphedema ( *p* = 0.002).

**Table 2:** Final univariate logistic regression model for the development of adhesive capsulitis in breast cancer patients

Variables	Case	Control	OR	95% CI		*p*
				Inferior	Superior	
Sociodemographic data						
Age group						
< 60 years	33	72	1.00			
≥ 60 years	6	34	2.60	0.99	6.79	0.052
BMI						
Normal	6	40	1.00			
Overweight	14	36	1.63	0.70	3.79	0.257
Obesity	19	30	4.22	1.50	11.86	0.006
Race						
White	16	36	1.35	0,64	2.88	0.432
Not white	23	70	1.00			
Marital status						
Married	18	48	0.95	0.39	2.31	0.918
Divorced	6	16	0.95	0.30	3.07	0.938
Widow	4	14	0.73	0.20	2.70	0.634
Single	11	28	1.00			
Monthly income						
None	2	10	1.00			
Up to 1 MW	16	56	1.43	0.28	7.19	0.665
2–3 MW	18	37	2.43	0.48	12.28	0.282
≥ 4 MW	3	3	5.00	0.55	45.39	0.153
Education						
Illiterate	1	5	1.00			
Elementary school	5	22	1.14	0.11	11.99	0.915
Middle school	6	16	1.87	0.18	19.53	0.599
Highschool	26	51	2.55	0.28	22.97	0.404
Higher	1	12	0.42	0.02	8.05	0.562
Dominance						
Right-handed	36	101	0.59	0.14	2.61	0.491
Left-handed	3	5	1.00			
Clinical breast data						
Affected breast						
Right	18	52	0.46	0.09	2.26	0.341
Left	18	50	0.48	0.10	2.36	0.366
Both	3	4	1.00			
Number of biopsies						
1	12	50	0.54	0.14	2.05	0.366
2 to 3	23	47	1.10	0.31	3.96	0.883
≥ 4	4	9	1.00			
Breastfeeding						
Yes	36	90	2.13	0.59	7.77	0.250
No	3	16	1.00			
Menopause						
Yes	30	82	0.98	0.41	2.34	0.956
No	9	24	1.00			
Chemotherapy						
Yes	33	89	1.05	0.38	2.89	0.924
No	6	17	1.00			
Radiotherapy						
Yes	35	71	4.31	1.42	13.10	0.010
No	4	35	1.00			
Endocrinotherapy						
Yes	25	65	1.13	0.53	2.41	0.760
No	14	41	1.00			
Previous hormone replacement therapy						
Yes	1	10	0.25	0.03	2.04	0.197
No	38	96	1.00			
Breast cancer stage						
Initial illness	23	60	1.00			
Advanced disease	16	46	1.10	0.52	2.32	0.798
Surgery						
Yes	38	80	12.35	1.62	94.44	0.015
No	1	26	1.00			
Type of surgery						
Quadrantectomy	18	39	1.00			
Mastectomy	20	41	1.06	0.49	2.29	0.888
Lymph node dissection						
No	5	15	1.00			
Sentinel	9	23	1.17	0.33	4.19	0.805
Axillary dissection	24	42	1.71	0.55	5.30	0.350
Immobilization						
Yes	22	26	3.98	1.84	8.62	<0.001
No	17	80	1.00			
Immobilization time						
1 to 3 Weeks	8	11	1.00			
≥ 4 Weeks	14	15	1.28	0.40	4.12	0.675
Lymphedema						
Yes	13	8	4.68	1.74	12.61	0.002
No	25	72	1.00			

**Abbreviations:** BMI, Body Mass Index; CI, confidence interval; MW, minimum wages; n, absolute frequency; OR, odds ratio; SD, standard deviation.

Multivariate logistic regression is demonstrated in Table 3 . Shoulder immobilization (OR = 3.13; 95% CI: 1.30–7.52; *p* = 0.011), lymphedema (OR = 4.60; 95% CI: 1.59–13.28; *p* = 0.005), and obesity (OR = 3.91; 95% CI: 1.27–12.01; *p* = 0.017) showed statistically significant association with the AC outcome.

**Table 3:** Final multivariate logistic regression model for the development of adhesive capsulitis in breast cancer patients

Variables	OR	95% CI		*p*
		Inferior	Superior	
Age group				
≥ 60 years old	2.48	0.76	8.08	0.132
BMI				
Obesity	3.91	1.27	12.01	0.017
Monthly income				
≥ 4 SM	3.64	0.28	47.64	0.325
Radiotherapy				
Yes	1.21	0.28	5.32	0.801
Previous hormone replacement therapy				
Yes	5.11	0.41	64.01	0.206
Surgery				
Yes	8.20	0.99	68.01	0.059
Immobilization				
Yes	3.13	1.30	7.52	0.011
Lymphedema				
Yes	4.60	1.59	13.28	0.005

**Abbreviations:** BMI, body mass index; CI, confidence interval; OR, odds ratio.

## Discussion

We investigated predictor factors for AC as outcome in patients undergoing treatment for BC. Predictive variables were obesity, shoulder immobilization, and lymphedema. Our initial hypothesis of radiation therapy and mastectomy associated with the outcome was not verified. The hypothesis was considered partially accepted, as shoulder immobilization was associated with the outcome.

Obesity is a world health issue correlating with several organic dysfunctions, including upper extremity pain. 12 It is part of the metabolic syndromes, a set of risk factors for cardiovascular outcomes and type 2 diabetes mellitus. 13 The latter are established predictors for the development of AC, as well as dyslipidemia. 1 3 In agreement with Kingston et al., 2 our study verified an association between obesity and AC. Thus, women diagnosed with BC must be instructed to manage body weight during treatment to prevent this shoulder condition.

Glenohumeral joint immobilization has been reported in the literature as a risk factor for AC, 14 15 which has also been verified in our study. Furthermore, Recchia et al. showed significant correlation between upper extremity morbidity and a decrease in quality of life after BC treatment. 16 Thus, the recommendation for BC patients following surgery should be a short splinting period followed by early movement of the shoulder. This would lead to improvement in shoulder pain and mobility. 17

A systematic review by Yang et al. concludes that implementing an exercise program before BC surgery focusing on shoulder motion may benefit ipsilateral upper extremity recovery. 18 In a study of BC survivors, Reigle and Zhang point out that patients who adhere to a postsurgery rehabilitation program may improve their functional abilities. 19

However, no association between immobilization time and the development of AC was verified in women with BC, which is not in agreement with other studies demonstrating this relation. 17 20 21 Only 48 patients in our study underwent immobilization and were consequently analyzed under variable “immobilization time.” This accounted for 33.1% of the total sample (22 cases and 26 controls) and only 14 cases underwent splitting for ≥4 weeks. This small subgroup may have favored a type II error of false-negative findings.

Another significant predictive factor for the development of AC in BC was lymphedema. In our study, 34.2% of the case group patients had previous history of lymphedema, in agreement with literature findings of approximately 20% BC-related lymphedema patients presenting with ipsilateral AC. 22 23 The therapy for this type of cancer directly involves all neuromusculoskeletal shoulder tissues, which accounts for the reason why BC survivors develop weakness, fatigue, reduced motion, and lymphedema.

This damage to the shoulder complex may be associated with diagnoses such as AC and postmastectomy syndrome. 9 24 Lymphedema does not usually present with pain but it is often correlated with various inflammatory processes that are related either to surgery or to other BC treatments, which could contribute to the development of several shoulder conditions, such as AC. 22

It is important to note that our study's objective was to find the association between predictor variables in women treated for BC and AC development. Oncologists seeing such patients should be able to recognize potential associated factors, diagnose AC with no delay, and refer them to specialized treatment. Orthopedists, on their end, should be aware of AC as one of the complications for BC treatment and use the therapy methods available to improve quality of life for affected women. 25 Patients under treatment for BC must be encouraged to exercise their shoulders after surgical procedures, be it in physiotherapy or hydrotherapy sessions, especially obese patients who developed lymphedema during treatment.

Despite our contribution to the clarification of AC in patients under treatment for BC, this study has some limitations. It is an analytical case-controlled study, and, as such, susceptible to a memory bias. Due to its design, it cannot investigate any cause and effect relationship. Furthermore, nonprobabilistic sampling at a BC-treatment specialty center may have imposed a selection bias, which did not allow for all patients presenting with the condition to be included in the study.

However, using a control group of BC patients without AC allowed for the comparison of the obtained data, aiding in improving scientific knowledge of this incapacitating joint condition's development in patients who are already facing the burden of a malignant condition. Additionally, the sampling was conducted with a minimum of two controls for each eligible case.

## Conclusion

Shoulder immobilization, lymphedema, and obesity are predictive factors for the development of AC in women with BC.
